# Genome-wide comparative analysis reveals selection signatures for reproduction traits in prolific Suffolk sheep

**DOI:** 10.3389/fgene.2024.1404031

**Published:** 2024-06-07

**Authors:** Hua Yang, Mengting Zhu, Mingyuan Wang, Huaqian Zhou, Jingjing Zheng, Lixia Qiu, Wenhua Fan, Jinghui Yang, Qian Yu, Yonglin Yang, Wenzhe Zhang

**Affiliations:** ^1^ State Key Laboratory of Sheep Genetic Improvement and Healthy Production, Xinjiang Academy of Agricultural and Reclamation Science, Shihezi, China; ^2^ College of Animal Science, Xinjiang Agricultural University, Urumqi, China; ^3^ College of Animal Science and Technology, Shihezi University, Shihezi, China

**Keywords:** selection signatures, fst, reproduction traits, candidate genes, sheep

## Abstract

The identification of genome-wide selection signatures can reveal the potential genetic mechanisms involved in the generation of new breeds through natural or artificial selection. In this study, we screened the genome-wide selection signatures of prolific Suffolk sheep, a new strain of multiparous mutton sheep, to identify candidate genes for reproduction traits and unravel the germplasm characteristics and population genetic evolution of this new strain of Suffolk sheep. Whole-genome resequencing was performed at an effective sequencing depth of 20× for genomic diversity and population structure analysis. Additionally, selection signatures were investigated in prolific Suffolk sheep, Suffolk sheep, and Hu sheep using fixation index (*F*_ST_) and heterozygosity *H*) analysis. A total of 5,236.338 Gb of high-quality genomic data and 28,767,952 SNPs were obtained for prolific Suffolk sheep. Moreover, 99 selection signals spanning candidate genes were identified. Twenty-three genes were significantly associated with KEGG pathway and Gene Ontology terms related to reproduction, growth, immunity, and metabolism. Through selective signal analysis, genes such as *ARHGEF4*, *CATIP*, and *CCDC115* were found to be significantly correlated with reproductive traits in prolific Suffolk sheep and were highly associated with the mTOR signaling pathway, the melanogenic pathway, and the Hippo signaling pathways, among others. These results contribute to the understanding of the evolution of artificial selection in prolific Suffolk sheep and provide candidate reproduction-related genes that may be beneficial for the establishment of new sheep breeds.

## 1 Introduction

China has the largest sheep flock and is the largest producer of sheep meat worldwide. According to the “National Breed List of Livestock and Poultry Genetic Resources” (2021 edition), there are 89 sheep breeds in China, including 44 indigenous breeds, 32 improved breeds, and 13 introduced breeds. Developing sheep breeds with high prolificacy has become a key goal in livestock breeding (Cottle, 2010; Miao et al., 2016; Gowane et al., 2017). Many new sheep breeds have been established in China in the past decade, such as Luxi Black Head sheep (Liu et al., 2022), Huang-huai sheep (Quan et al., 2020), and a new breed of prolific Suffolk sheep (Wang et al., 2020), which was established through grading hybridization between Suffolk sheep and Hu sheep over 12 years of breeding. Suffolk sheep make excellent male parents for terminal crosses while Hu sheep are known for their high fertility, and are widely used in sheep farming in Xinjiang, China. Thus, prolific Suffolk sheep have the advantages of high-quality meat from Suffolk sheep and prolificacy characteristics from Hu sheep.

Selection signature analysis can help identify the genomic imprint of livestock resulting from the process of domestication or artificial selection. Based on this strategy, researchers can scan the regions that are associated with important economic traits that have been subjected to selection during the domestication of livestock, locate the selected genes or genetic markers, and identify the genetic mutations associated with these traits, so as to achieve variety improvement and new germplasm creation (Horscroft et al., 2019). Additionally, population-based resequencing can reveal the evolutionary relationships between populations, help identify the excellent genetic resources of each breed, and contribute to the understanding of the genetic diversity between populations; this provides strong support for the selection of new breeds and the promotion of the development of animal husbandry. Li et al. conducted 25.7× whole-genome resequencing of wild and domestic sheep, which revealed the genetic mechanism underlying various agricultural traits in domesticated sheep (e.g., reproduction, wool production), thereby providing valuable genomic resources for research on sheep genetics (Li et al., 2020). Similarly, Zhang et al. employed genome resequencing technology to uncover the natural selection molecular imprinting of wild and domesticated sheep. They identified *IFI44, PNK2*, and *RNF24* as being related to the immunity of sheep, thereby providing insights into the molecular mechanism underpinning the origins of phenotypic variation induced by sheep domestication and improvement (Zhang et al., 2022). Sweet-Jones et al. used whole-genome resequencing to perform high-depth scanning for selection signatures linked to the adaptability of Welsh sheep (Sweet-Jones et al., 2021). The authors reported that the *RNF24, PANK2*, and *MUC15* genes had strong selection signals, with potential functions in the environmental adaptability of local Welsh breeds. Furthermore, Wang et al. (2017) sequenced mixed pools of multiple-lamb and single-lamb Duolang sheep populations, and identified six genes related to reproductive performance, including *INHBA, NCOA1, INGS, BMPR-IB, ARNT*, and *KLHL1* (Sui, 2017). Zhang et al. used fixation index (*F*_ST_) analysis to detect genome-wide selection signals in five sheep breeds, and found that *RXFP2, GHR, and ASIP* were related to the shape, growth, and lipid metabolism of horns (Zhang et al., 2013). Therefore, we used whole genome resequencing technology to screen genes related to important economic traits, reveal the genetic basis of breeding breeds, and provide a basis for the selection and breeding of multiple new breeds of mutton sheep.

In this study, we resequenced the whole genomes of 90 Hu sheep, Suffolk sheep, and prolific Suffolk sheep to explore their genetic structure and the genetic variance among the breeds as well as identify candidate regions and genes related to reproductive traits. Additionally, *F*_ST_ and *H* analysis was used to identify selection signals unique to prolific Suffolk sheep, while functional enrichment analysis was undertaken to identify major genes closely related to reproductive traits. Our aim was to provide a theoretical basis for the breeding of prolific Suffolk sheep breeds as well as offer further insights into the selection of local sheep breeds in China.

## 2 Materials and methods

### 2.1 Sample collection

**FIGURE 1:**
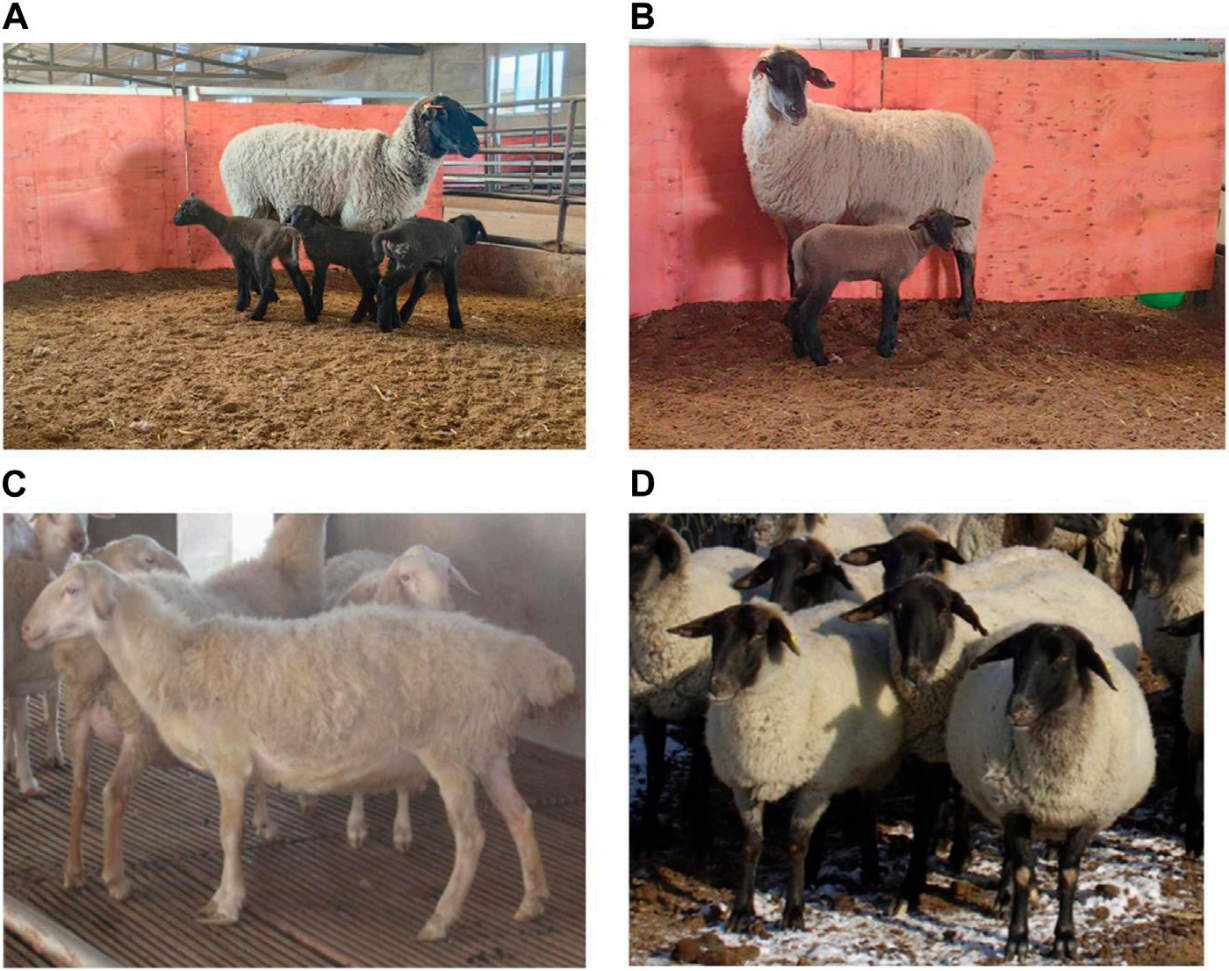
Sheep breeds. **(A)** Prolific Suffolk sheep in the multi-lamb group. **(B)** Prolific Suffolk sheep in the single-lamb group. **(C)** Hu sheep. **(D)** Suffolk sheep.

In this study, based on pedigree information, 50 healthy and unrelated prolific Suffolk sheep of the same age (26 rams and 24 ewes with 196% average lambing rate) were selected. The 24 ewes were divided into two groups, namely, a multi-lamb group, consisting of 12 ewes with a 275% lambing rate (Figure 1A), and a single-lamb group, comprising 12 ewes with a lambing rate of 117% (Figure 1B). Twenty Hu sheep with a 230% lambing rate (Figure 1C) and 20 Suffolk sheep with a 140% lambing rate (Figure 1D) were also used (Supplementary Table S6). All the sheep came from the sheep farm of Xinjiang Academy of Agricultural and Reclamation Science. Blood samples (5 mL) collected from the jugular vein were placed in EDTA-Na_2_ and stored at −20 °C until further processing.

### 2.2 DNA isolation and sequencing

DNA was extracted from blood using the TIANamp Blood DNA Kit (TIANGEN, China) according to the manufacturer’s instructions. The quality of the genomic DNA was assessed by 1% agarose gel electrophoresis while its concentration and purity were evaluated using a NanoDrop 2000 spectrophotometer. DNA libraries were prepared using the TruSeq Library Construction Kit. The DNA was randomly fragmented to an average size of 350 bp using ultrasonication and sequencing libraries were constructed following the manufacturer’s instructions (Illumina, San Diego, CA, USA). The libraries were paired-end sequenced at ∼20× coverage on the Illumina HiSeq2500 Platform (Illumina Inc.) by Beijing Compass Biotechnology Co., Ltd (Beijing, China). To provide reliable data for subsequent analysis, the original sequencing data were filtered to remove reads with linker sequences, sequences with more than 10% N content, and low-quality data (Q-value ≤5). Subsequent analyses were based on these clean data.

### 2.3 Read alignment and variable annotation

The effective sequencing data were compared to the sheep reference genome (Oar_v4.0) through BWA (v.0.7.12) with the parameters “mem -t 4 -k 32 -M” (Li and Durbin, 2009). Individual SNPs were detected with SAMtools (v.1.2) using the parameters “mpileup -m 2 -F 0.002 -d 1,000” (Li et al., 2009). The filtering criteria for minimizing variant calling errors were as follows: variant sites with QD < 2.0, MQ < 20, and FS > 60.0 were discarded and the remaining variants were annotated using ANNOVAR v.21-Jun-2013.

### 2.4 Phylogenetic analysis and population Dynamics

Based on the neighbor-joining (NJ) method, a phylogenetic tree was constructed with the set of quality-filtered SNPs using the Phylogeny Inference Package (PHYLIP) (Felsenstein, 1989). Cluster analysis for elucidating population structure was performed using ADMIXTURE (v.1.3.0) with the following parameters: “for *K* in 2 3, do admixture --cv sheep.bed $K | tee log${K }.out, done” with a maximum of 10,000 iterations. Principal Component Analysis (PCA) of the 90 samples was performed using the EIGENSOFT package, v.7.2.1 (Price et al., 2006).

### 2.5 Selective sweep analysis

In this study, *H* and the *F*_ST_ were calculated with VCFtools v.0.1.14 using a sliding window approach (100-kb windows with a 50-kb step size) (Zhang et al., 2021). The parameters for the VCFtools program were as follows: “--fst-window -size 100,000 --fst-window-step 50,000”. The top 5% *F*_ST_ and *H* values were selected as the threshold to map the selected loci on autosomes and identify differences between any two populations. The intersection candidate area was considered as the selection signal in the test.

The *F*_ST_ calculation formulas:

$F_{\text{ST}}=\frac{H_{T}-H_{S}}{H_{T}}$ , among which H_T_ represents the expected heterozygosity of alleles in the total population, and H_S_ represents the weighted average heterozygosity of different subgroups in the total population.

The *H* calculation formulas:

$\text{He}=1-\sum {\text{pi}}^{2}$ , among which Pi is the frequency of the *i*th allele, and the expected heterozygosity of the sliding window is the mean value of the expected heterozygosity of each SNP site in the region.

### 2.6 Functional enrichment analysis

Gene Ontology (GO) and Kyoto Encyclopedia of Genes and Genomes (KEGG) pathway enrichment analysis was performed to identify clusters of functionally related genes (Harris et al., 2004; Jiao et al., 2012). GO term analysis included the biological process (BP), molecular function (MF), and cellular component (CC) categories. A significance threshold of <0.05 was used to determine GO term enrichment in a set of genes. KEGG pathway enrichment analysis was performed using KOBAS 2.0 (http://kobas.cbi.pku.edu.cn/) and a corrected *p*-value of <0.05 was set as the threshold for significance. In order to avoid false positive results, the enrichment analysis result P was corrected by multiple tests (False Discovery Rate, FDR).

The formula of FDR is:

$FDR=P\times \frac{n}{\left(\text{rankP}\right)}$ , among which, P is the original *p*-value, n is the number of tests, and rankP is the level of a specific original *p*-value. When FDR ≤0.05, GO terms and pathways that meet this condition are defined as significant enrichment of candidate genes.

## 3 Results

### 3.1 Sequencing and mapping

A total of 5,245.855 Gb of raw data for 90 individuals were obtained by resequencing on the Illumina HiSeq 2,500 platform, with 5,236.338 Gb of clean reads remaining after filtering. The Q20 value of the clean reads was ≥95.73% while the Q30 value was ≥89.69%. The GC content ranged between 43.09% and 46.62%. The genome mapping rate relative to the sheep reference genome (Oar_v4.0) ranged between 98.65% and 99.32%. The average coverage depth was approximately 17.39× for all three sheep breeds, the 1 × average coverage was more than 98.06%, and the 4 × average coverage was more than 94.29%, indicating that the data were accurate and reliable (Supplementary Table S1). SAMTools was used to collect summary information from the input binary alignment/map (BAM) files, compute the likelihood of each genotype, and then convert the information into binary variant call format (BCF). ANNOVAR software was used for the functional annotation of gene mutations and for converting the data into variant call format (VCF) for subsequent analysis.

### 3.2 SNP identification and annotation

Additionally, 84,505,591 SNPs in all three sheep breeds and 28,767,952 SNPs in prolific Suffolk sheep were annotated with SAMTools v.0.1.19. In the prolific Suffolk sheep population, 204,304 exonic SNPs, 1,520,657 non-synonymous mutations (5.29%), and 2,101,043 synonymous mutations were identified. In the Hu sheep population, a total of 29,080,742 SNPs were annotated, 207,059 of which were located in exons; 620,064 non-synonymous mutations (2.13%) and 866,996 synonymous mutations were also identified. In Suffolk sheep, a total of 26,656,897 SNPs were annotated; 187,316 of these SNPs were located in exons, while 606,196 non-synonymous mutations (2.27%) and 838,500 synonymous mutations were identified. The non-synonymous/synonymous ratio was 0.72 in all three sheep breeds (Supplementary Table S2).

### 3.3 Phylogenetic analysis

**FIGURE 2:**
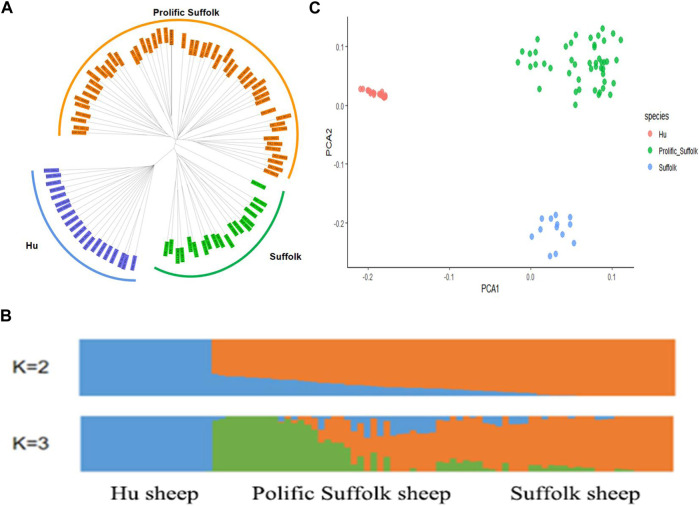
Population genetics analysis. **(A)** Neighbor-joining phylogenetic tree. **(B)** Population structure based on 90 individuals as determined using ADMIXTURE with *K* = 2, 3. **(C)** The results of the Principal Component Analysis (PCA) for the three sheep breeds.

To investigate the genetic relationship among the three sheep breeds, a genetic distance matrix was calculated based on the SNPs after whole-genome quality control, and a phylogenetic tree of the three populations was constructed using the NJ method. The NJ tree was constructed based on the JTT + G model with 1,000 bootstrap replicates. NJ tree analysis showed that the three sheep varieties were separated into three independent genetic groups (Figure 2A).

### 3.4 Population genetic Structure

As shown in Figure 2B, at *K* = 2, Hu sheep clustered into one type, prolific Suffolk sheep and Suffolk sheep clustered into another type, and there was gene flow within them. At *K* = 3, prolific Suffolk sheep were clearly separated from both Hu and Suffolk sheep. Prolific Suffolk sheep and Suffolk sheep were clustered together. Hu sheep had a long genetic distance from the other two populations. Also, a close genetic relationship was discovered between prolific Suffolk sheep and Suffolk sheep, which was also consistent with the breeding process of the former.

### 3.5 Principal component analysis

To examine the genetic relationship among and within the three varieties, we conducted a PCA. The first Eigenvector clearly distinguished Hu sheep from Prolific Suffolk sheep and Suffolk sheep, while the second Eigenvector distinguished prolific Suffolk sheep from Suffolk sheep (Figure 2C). As expected, the results of the PCA were similar to those obtained for the phylogenetic tree and population genetic structure analysis, showing that the selected samples had good consistency.

### 3.6 Selective imprints of prolific Suffolk sheep, Suffolk sheep, and Hu sheep

**FIGURE 3:**
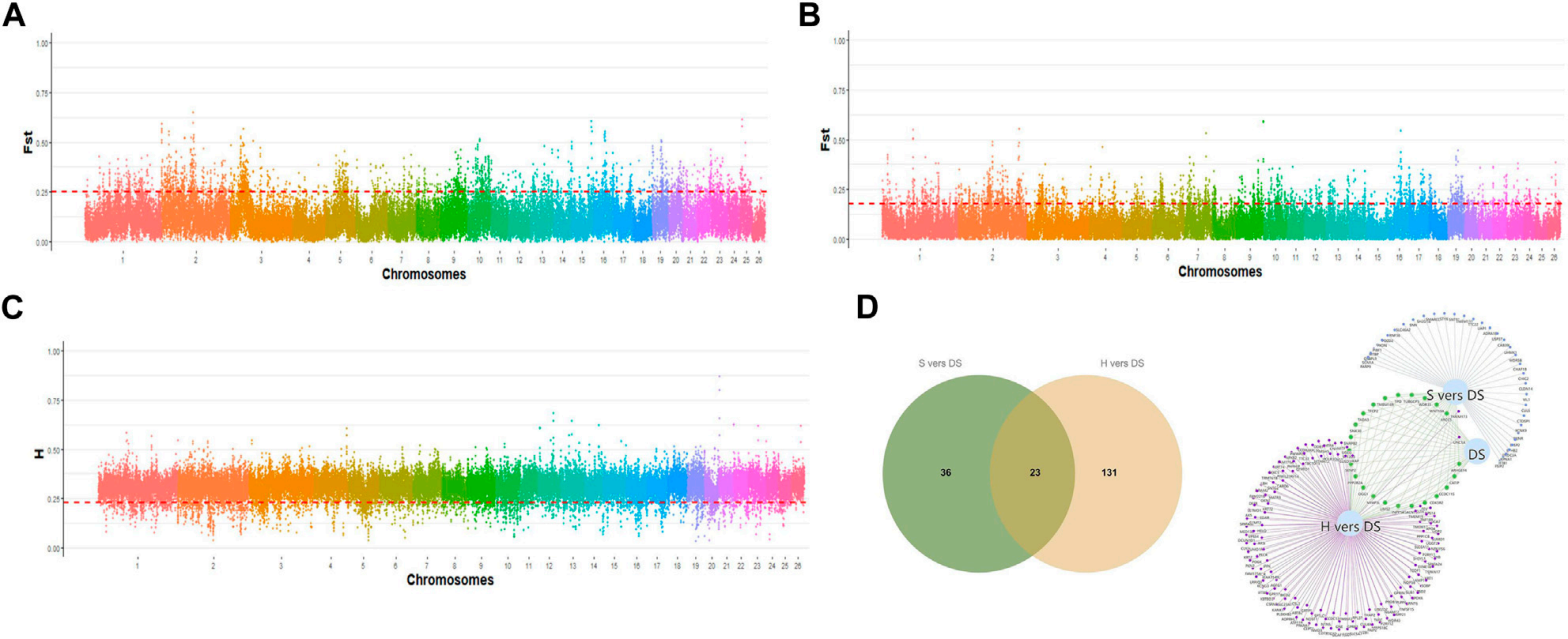
**(A)** The distribution of *F*_ST_ values on autosomes in prolific Suffolk sheep and Hu sheep. **(B)** The distribution of *F*_ST_ values on autosomes in prolific Suffolk sheep and Suffolk sheep. **(C)** The distribution of heterozygosity (*H*) on autosomes in prolific Suffolk sheep. **(D)** Venn diagram of the unique genes in prolific Suffolk sheep.

To accurately identify the biological markers associated with the germplasm characteristics of prolific Suffolk sheep, the sliding window method (window size: 100 kb, step size: 50 kb) was used to scan the selection signals on autosomes. The top 5% *F*_ST_ and *H* values were selected as the threshold to map the selected loci on autosomes and identify differences between any two populations. A total of 137 selected regions were scanned and 154 candidate genes were mapped in the comparison between prolific Suffolk sheep and Hu sheep (*F*_ST_ > 0.249001 and *H* < 0.224707) (Figures 3A,C; Supplementary Tables S3 and S4). For prolific Suffolk sheep *versus* Suffolk sheep, a total of 99 selected regions were screened and 59 candidate genes were mapped (*F*_ST_ > 0.178916 and *H* < 0.224707) (Figures 3B,C; Supplementary Tables S3 and S4). Repeats were removed from the 213 candidate genes in the two comparison groups. Finally, 190 candidate genes were screened, 14 of which were related to reproduction traits, including *WNT10A*, *SENP2*, and *WNT6* (Table 1). Furthermore, 23 genes, including *ARHGEF4*, *CATIP*, *CCDC115*, and *CDK5R2*, were found to be unique to prolific Suffolk sheep (Figure 3D; Supplementary Table S5). These genes may represent the germplasm-specific genetic information retained during prolific Suffolk sheep breeding and selection (Table 2).

**TABLE 1:** Enriched items and genes related to reproduction in prolific Suffolk sheep.

Term	Count	Gene	FDR
KEGG: Wnt signaling pathway	3	WNT10A, SENP2, WNT6	0.012556028
KEGG: Circadian entrainment	1	PRKAA1	0.04687154
KEGG: Oocyte meiosis	2	SPDYA, PPP1CB	0.012556028
KEGG: Insulin resistance	2	PPP1CB, PRKAA1	0.012556028
GO: Prostaglandin biosynthesis	1	CD74	0.04687154
GO: Positive regulation of gene expression	1	KANK1	0.04687154
GO: Negative regulation of oocyte maturation	1	SHB	0.04687154
GO: Intrauterine embryonic development	1	CUL4A	0.022834044
GO: Sperm development	1	FNDC3A	0.04687154
GO: Spermatogenesis	3	IFT81, PIWIL2, PAIP2	0.04687154
GO: Oogenesis	1	PIWIL2	0.04687154
GO: Regulation of circadian rhythm	1	PRKAA1	0.04687154

**TABLE 2:** Genetic variation information for prolific Suffolk sheep.

CHR	Gene	SNP physical location
1	SENP2	199,647,970, 199,655,961
2	ARHGEF4	114,458,386
2	CCDC115	113,068,460, 113,068,647
2	FEV	219,905,212
2	MFAP3L	110,044,500, 110,059,546, 110,059,801, 110,059,990, 110,060,096
2	PPP2R2A	39,204,255
2	CATIP	219,363,185, 219,363,553, 219,364,173, 219,364,174
2	SFT2D3	116,650,526, 116,650,551
2	SNX30	10,873,818, 10,885,812, 10,885,813
2	TFCP2	185,493,910, 185,500,093, 185,547,870, 185,547,984
2	TPO	719,199, 719,200, 719,210, 719,221, 722,337, 724,813, 724,819, 726,979, 748,143
2		
2	WDR33	116,584,306, 116,588,784, 116,597,221, 116,597,328, 116,597,342, 116,629,864, 116,629,945
2	XRCC5	217,112,784, 217,115,691, 217,115,696, 217,115,780, 217,133,944, 217,176,443
2	CDK5R2	219,882,633, 219,882,634
2	GALNTL6	107,356,681
2	LIMS2	116,728,390, 116,728,516, 116,728,884, 116,729,018, 116,729,124, 116,738,675, 116,738,777, 116,738,830
2	TMEM169	217,095,393, 217,095,612
2	TUBGCP5	112,982,203, 112,985,801, 112,987,176, 113,001,713, 113,013,684, 113,034,954
2	WNT10A	219,838,113, 219,838,119
13	SNRPB2	10,045,752
19	OGG1	16,885,800, 16,886,615, 16,892,224, 16,893,120, 16,893,156
19	TADA3	16,853,617, 16,859,465
22	INPP5A	50,041,342, 5,0,041,821, 50,047,784, 50,048,122, 50,048,133, 50,048,234, 50,048,253, 50,048,344, 50,048,365, 50,048,407, 50,048,478, 50,048,503, 50,048,560, 50,048,649, 50,051,633, 50,092,909, 50,096,284, 50,096,399, 50,143,465

### 3.7 Functional enrichment analysis

**FIGURE 4:**
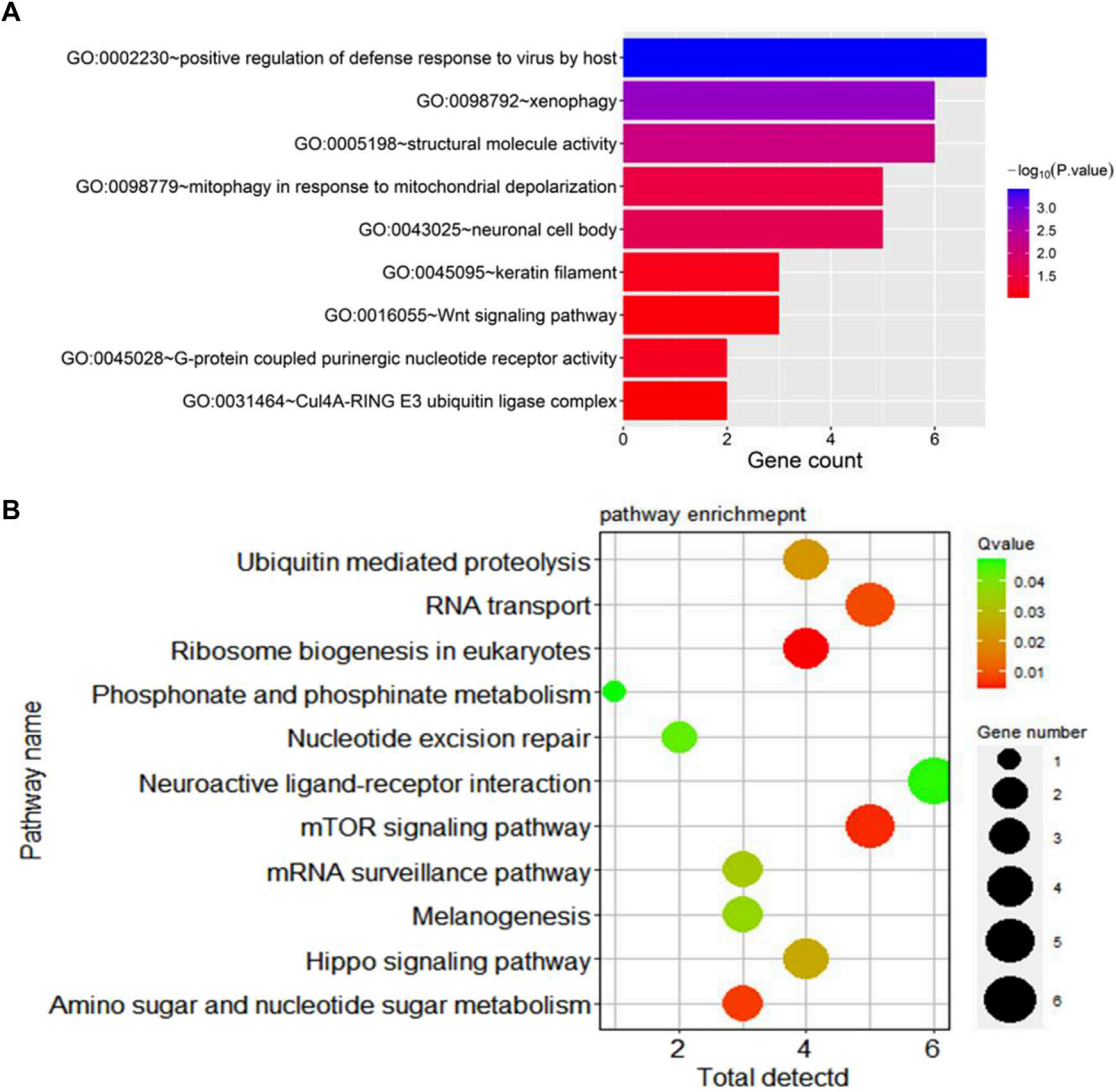
Enrichment results for specific genes in prolific Suffolk sheep. **(A)** GO term enrichment. **(B)** KEGG pathway enrichment.

Additionally, Gene Ontology (GO) enrichment analysis revealed key biological processes associated with germplasm-specific genes in prolific Suffolk sheep. Candidate genes were enriched in positive regulation of defense response to virus by host, and G protein-coupled purinergic nucleotide receptor activity and other processes and were mainly related to signal transduction. Meanwhile, KEGG pathway enrichment analysis indicated that the candidate genes *WNT10A, PPP2R2A*, and *WDR33* were enriched in the mTOR signaling pathway, melanogenesis, and the Hippo signaling pathway (*p* < 0.05) (Figure 4).

### 3.8 Selective imprints of reproduction traits in prolific Suffolk sheep

**FIGURE 5:**
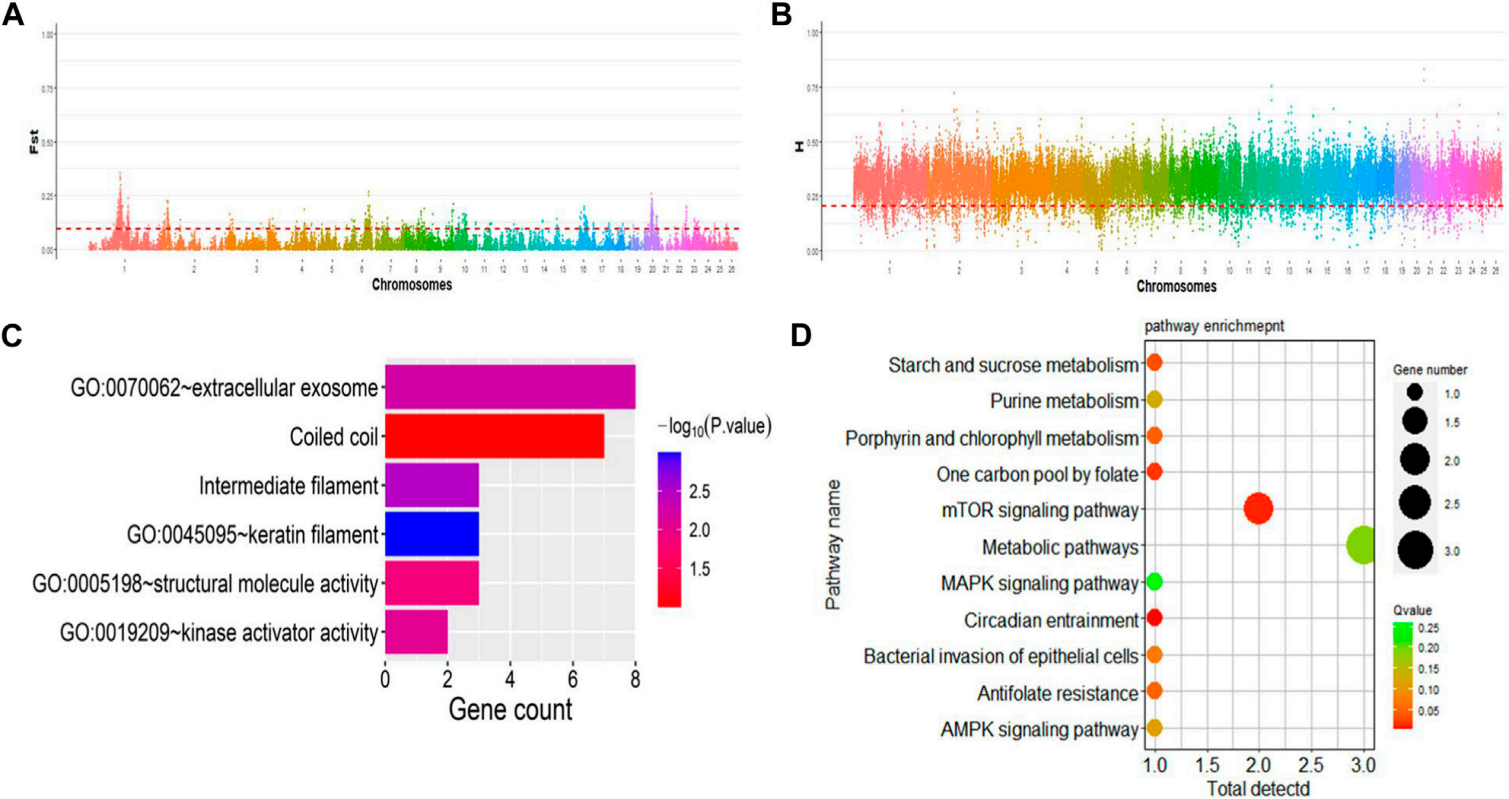
Selective imprints of the reproductive traits in multi-lamb and single-lamb groups. **(A)** Selection signals in the multi-lamb and single-lamb groups. **(B)** Distribution of heterozygosity (*H*) on the autosome of the multi-lamb group. **(C)** GO term enrichment analysis for genes related to reproductive traits. **(D)** KEGG pathway enrichment analysis for genes related to reproductive traits.

Next, we explored the selective imprints of reproduction traits in prolific Suffolk sheep resulting from artificial selection. For this, selection signals were compared between the multi-lamb and single-lamb groups using 100-kb sliding windows with a step size of 50 kb across the genome. A combination of both *F*_ST_ and *H* analysis methods was also employed to scan for selection signals on autosomes. We identified 29 selected regions and 24 candidate genes (Figures 5A,B; Table 3). Genes related to reproductive traits, such as *MTNR1A, ITSN1*, and *GBE1*, among others, were subjected to GO and KEGG enrichment analysis and were found to be associated with litter size. Furthermore, the identified genes were mainly enriched in circadian entrainment, MAPK signaling pathway, AMPK signaling pathway, and mTOR signaling pathway (Figures 5C,D).

**TABLE 3:** Variation information for genes in prolific Suffolk sheep.

CHR	Gene	SNP physical location	Function
1	ADAMTS5	127,446,705, 127,446,839, 127,447,076, 127,447,184, 127,472,912, 127,472,921, 127,482,912, 127,484,992, 127,485,082, 127,485,157, 127,488,111, 127,495,988	Energy metabolism
1	DONSON	120,015,059	Reproduction
1	DUSP27	117,131,442, 117,190,778, 117,191,119, 117,191,316, 117,191,349, 117,191,445, 117,191,529, 117,191,628, 117,191,743, 117,191,816, 117,192,087, 117,192,525, 117,192,657, 117,192,823	Metabolism
1	GART	120,073,957, 120,074,050	Purine metabolism
1	GBE1	1,47,518,295, 1,47,520,159, 1,47,520,174, 147,563,933, 147,577,172, 147,577,187, 147,582,028, 147,683,665, 147,723,202	Reproduction
1	HUNK	1,21,618,954, 1,21,619,011, 1,21,619,040, 121,646,603, 121,646,696, 121,749,125	Metabolism
1	ILDR2	116,851,460, 116,852,804	Immunity and adaptability
1	ITSN1	119,732,240, 119,735,835, 119,735,886, 119,782,548, 119,816,398	Reproduction
1	OLIG2	120,627,151, 120,627,152, 120,627,540, 120,627,668, 120,629,854, 120,630,177	Growth and development
1	SON	120,015,059	Immunity and adaptability
3	KRT1	133,403,474, 133,404,568	Woolly hair
3	KRT2	133,430,103, 133,433,319, 133,435,631, 133,437,302, 133,437,345, 133,437,386	Woolly hair
3	KRT72	133,462,372, 133,464,780, 133,467,935, 133,472,160, 133,472,188, 133,472,212, 133,472,242, 133,473,817, 133,473,983	Woolly hair
3	KRT74	133,485,704, 133,485,900, 133,485,980, 133,486,008, 133,486,034, 133,486,870, 133,488,025, 133,492,003, 133,492,044	Woolly hair
6	LAMTOR3	69,999,643, 69,999,944, 69,999,951	Reproduction
8	TBC1D32	16,875,389, 16,890,269, 16,890,271, 16,980,424, 16,980,503, 16,995,212, 17,006,420, 17,017,115, 17,018,856, 17,018,917, 17,029,097	Immunity and adaptability, reproduction
10	CAB39L	19,271,752, 19,271,767, 19,271,770, 19,271,800	Reproduction
17	HECTD4	61,559,479, 61,559,548, 61,559,617, 61,559,769, 61,564,204, 61,568,425, 61,581,290, 61,581,291, 61,596,568, 61,615,697, 61,618,000, 61,622,821, 61,634,588, 61,634,683, 61,652,374, 61,653,187, 61,653,543, 61,656,956, 61,657,049, 61,657,362, 61,657,875, 61,657,899, 61,657,926, 61,657,971, 6,1,661,760, 6,1,661,991, 6,1,662,093, 61,664,342, 61,665,879, 61,665,918, 61,667,268, 61,667,478, 61,671,616, 6,1,671,723	Immunity and adaptability
20	CD2AP	20,634,974	Immunity and adaptability
22	DPYSL4	49,746,131, 4,9,752,001, 49,753,954, 49,753,979, 49,755,852, 49,757,414, 49,758,062, 49,760,159	Immunity and adaptability
22	EDRF1	44,115,409, 44,115,468, 44,118,262, 44,119,817, 44,119,856, 44,119,862, 44,124,147, 44,125,748, 44,132,651, 44,133,143	Immunity and adaptability
22	JAKMIP3	49,711,228, 49,711,258, 49,711,290, 49,711,320, 49,711,340, 49,711,341, 49,711,376, 4,9,711,775, 49,712,254, 49,720,952, 49,723,089, 49,723,111, 49,723,204	Growth and development
22	STK32C	49,763,343, 49,771,613, 49,774,512, 49,774,515, 49,774,906, 49,775,889, 49,775,907	Metabolism
26	MTNR1A	15,099,004, 15,099,204, 15,099,296, 15,099,314, 15,099,391	Reproduction

## 4 Discussion

Sheep farming plays an important role in the global animal husbandry industry, supplying a wide range of products such as wool, meat, milk, and skin. In China, sheep farming has been an important part of the agricultural economy and rural life for many centuries. Over recent years, the meat sheep breeding industry has undergone rapid development, characterized by an increasing abundance of germplasm resources, a gradually improving breeding system, and a steadily increasing level of sheep production (Meadows and Kijas, 2010). However, overall, there is still a large gap between China and developed countries in this respect, a limitation that can mostly be explained by the low fecundity of most Chinese breeds (Han et al., 2015). Litter size is a complex trait that is influenced by many factors, including genetic background, nutritional level, and feeding management. It is the main trait in sheep reproductive performance and one of the main selection objectives in meat sheep breeding programs (Baelden et al., 2005). Increasing litter size proved to be an effective means of improving the economic benefits of the mutton industry (Dong et al., 2013). Therefore, in this study, we performed genome-wide resequencing to analyze germplasm-specific genes, genetic variation, and the genome map of self-bred prolific Suffolk sheep. Furthermore, we identified the main genes and SNPs related to their reproductive traits. Our findings contribute to unraveling the germplasm characteristics and population genetic evolution of this new strain of Suffolk sheep and provide a basis for the creation of new breeds of prolific meat sheep in China.

Prolific Suffolk sheep is a new strain developed by our group through 12 years of breeding in Xinjiang, China. Hu sheep served as the female parents and Suffolk sheep as the male parents in hybrid breeding. The breeding process involved hybridization, fixation in a two-way crossbreed closed flock, and herd propagation, resulting in a breed with high prolificacy and high-quality meat performance. The new strain is a stabilized composite breed made up of 87.5% Suffolk blood proportion and 12.5% Hu blood proportion (Yang et al., 2021a). In this study, we found that the Hu sheep breed was highly distinct from the other two breeds, exhibiting a large genetic distance from both, which was also consistent with the breeding process of prolific Suffolk sheep. Through selective signal analysis, the *WNT10A, PPP2R2A*, and *WDR33* genes were found to be significantly related to reproductive traits in prolific Suffolk sheep and were highly associated with the mTOR signaling pathway, the melanogenic pathway, and the Hippo signaling pathway, among others. mTOR is a highly conserved serine-threonine protein kinase that forms two distinct complexes, mTORC1 and mTORC2 (Sui et al., 2021). mTOR complexes are sensitive to growth factor, amino acid, and oxidative stress stimulation and are involved in multiple biological processes, such as lipid metabolism, autophagy, protein synthesis, and nucleosome biosynthesis (Yao et al., 2021). The melanogenesis pathway involves a complex series of enzymatically and chemically catalyzed reactions (Pillaiyar et al., 2017). The MC1 receptor (MC1R) and its ligand melanocortin are important positive regulators of melanin production (Dnyane and Gadgil, 2020). MC1R activates cyclic AMP (cAMP) response element binding protein (CREB). Tyrosinase (TYR) is the rate-limiting enzyme in melanin synthesis, which occurs in specialized cell organelles, called melanosomes, which are transferred to keratinocytes through mechanisms that have not been fully characterized (Varghese et al., 2021). The results of the present study indicated that phenotypic uniformity was achieved in prolific Suffolk sheep during the 12 years of breeding. The appearance of pure black coat color on the head and limbs is one of the characteristics of the breed, indicating that differences in melanin production may underlie this phenotype. Additionally, the Hippo and WNT signaling pathways are closely related. The Hippo signaling pathway comprises a group of conserved kinases that can inhibit the normal growth of cells and participates in the regulation of organ and tissue size (Heallen et al., 2011; Yang et al., 2021b). The phosphorylation of the protein kinase Warts, a constituent of the Hippo signaling pathway, results in its activation, leading to a series of changes in signaling pathways associated with reproduction traits in female livestock (Vitezslav and Vladimir, 2018; Hidayah et al., 2019). The *WNT10A* gene has been shown to act in the canonical Wnt/β-catenin signaling pathway. It is expressed in epithelial and mesenchymal cells throughout tooth development and plays an important role in this process (Kanchanasevee et al., 2020; Zeng et al., 2021). In this study, we found that the selected genes were enriched in the WNT and mTOR signaling pathways, suggesting that *WNT10A* may have contributed to changes in tooth development, hair follicle growth, and reproductive performance during the breeding of prolific Suffolk sheep. Similarly, the *PPP2R2A* gene is mainly involved in signal transduction and may participate in the growth and development of mammals (Zhang et al., 2020). Cui et al. showed that the downregulation of *PPP2R2A* by CRISPR/Cas9-mediated deletion inhibits the growth of non-small cell lung cancer cells (Cui et al., 2020), which was similar to the results of this study. This suggests that the *WNT10A* and *PPP2R2A* genes may have played a role in the breeding process of prolific Suffolk sheep, affecting their growth and development, hair follicle growth, and reproductive performance.

Furthermore, selective imprint analysis indicated that the *MTNR1A* and *GBE1* genes were mainly involved in circadian entrainment, metabolism, and signal transduction, and were closely related to the high fecundity of prolific Suffolk sheep. In addition, MTNR1A has been reported to be related to sheep litter size (Luridiana et al., 2020; Stari et al., 2020). *MTNR1A* is a G protein-coupled seven-transmembrane receptor that mediates the effects of melatonin on mammalian circadian rhythms, thereby affecting mammalian reproductive function (Cosso et al., 2021). Notably, the *MTNR1A* gene is a key regulator of the reproductive traits of sheep and may be closely related to the litter size trait in prolific Suffolk sheep. He et al. analyzed the association between *MTNR1A* gene polymorphism and litter size in Small Tail Han sheep and showed that the mutation (TT) in the g.15118756C > T locus significantly increased litter size (He et al., 2012). Additionally, Starič et al. investigated the correlation between *MTNR1A* gene polymorphism and litter size in Slovenian sheep and similarly found that the g.17355452 locus had a significant effect on litter size (Stari et al., 2020). These results were similar to those recorded in this study. The protein encoded by the *GBE1* gene is a glycogen-branching enzyme that may be involved in energy regulation during animal growth (Li et al., 2018). UROIIIS, encoded by the *UROS* gene, may be involved in metabolic regulation during growth (Blouin et al., 2021). Our results suggested that the *GBE1* and *UROS* genes may regulate the growth, development, and metabolism of prolific Suffolk sheep.

Through selective signature analysis, we identified the genes that were specifically differentially expressed in prolific Suffolk sheep, a new strain developed by crossing Hu sheep and Suffolk sheep. These genes were mainly enriched in pathways related to reproduction, immunity, growth, energy metabolism, and sugar metabolism. Our findings provide both a basis for the molecular breeding of new breeds of prolific meat sheep as well as target genes and functional sites for the establishment of other new sheep breeds.

In conclusion, this study provided a comprehensive insight into the germplasm characteristics of prolific Suffolk sheep. We identified several germplasm-specific candidate genes and markers under selection in prolific Suffolk sheep, Suffolk sheep, and Hu sheep. These genes play essential roles in reproduction, growth and development, among other economic traits. The large number of genetic variants identified in the study represents an opportunity for further exploring the genetic diversity and the associated phenotypic variation in prolific Suffolk sheep. Our results contribute to the understanding of the genetic makeup of prolific Suffolk sheep and provide valuable information for future development and improvement of new breeds.

## Data Availability

The datasets presented in this study can be found in online repositories. The names of the repository/repositories and accession number(s) can be found in the article/Supplementary Material.

## Abbreviations

BP, Biological process; CC, Cellular component; GO, Gene Ontology; KEGG, Kyoto Encyclopedia of Genes and Genomes; MF, Molecular function; NJ, Neighbor-joining; PCA, Principal Component Analysis; VCF, Variant call format.
