# Supplementary material for: Genome-wide comparative analysis reveals selection signatures for reproduction traits in prolific Suffolk sheep
Source: Front Genet. 2024 Jun 7;15:1404031. doi: 10.3389/fgene.2024.1404031 (PMC11193351; doi:10.3389/fgene.2024.1404031)
Supplement: Supplementary file 1 [file DataSheet1.docx]

Supplementary Material

# Supplementary Tables

**Supplementary Table 1**. Detailed information on the sequencing and alignment statistics for the 90 Xinjiang native sheep individuals.

**Supplementary Table** **2**. Summary of SNPs in 90 sheep individuals.

**Supplementary Table** **3.** Details on putative regions experiencing selected sweeps (top 5% empirical cutoff) for *F*_ST_ values on each chromosome.

**Supplementary Table** **4.** Details on putative regions experiencing selected sweeps (top 5% empirical cutoff) for heterozygosity (*H*) values on each chromosome.

**Supplementary Table** **5.** Specific differentially expressed genes in prolific Suffolk sheep.
